# Non-face-to-face physical activity interventions in older adults: a systematic review

**DOI:** 10.1186/1479-5868-11-35

**Published:** 2014-03-10

**Authors:** Andre Matthias Müller, Selina Khoo

**Affiliations:** 1 Sports Centre, University of Malaya, 50603 Kuala Lumpur, Malaysia

**Keywords:** Media, Non-face-to-face interventions, Older adults, Physical activity, Systematic review

## Abstract

Physical activity is effective in preventing chronic diseases, increasing quality of life and promoting general health in older adults, but most older adults are not sufficiently active to gain those benefits. A novel and economically viable way to promote physical activity in older adults is through non-face-to-face interventions. These are conducted with reduced or no in-person interaction between intervention provider and program participants. The aim of this review was to summarize the scientific literature on non-face-to-face physical activity interventions targeting healthy, community dwelling older adults (≥ 50 years). A systematic search in six databases was conducted by combining multiple key words of the three main search categories “physical activity”, “media” and “older adults”. The search was restricted to English language articles published between 1^st^ January 2000 and 31^st^ May 2013. Reference lists of relevant articles were screened for additional publications. Seventeen articles describing sixteen non-face-to-face physical activity interventions were included in the review. All studies were conducted in developed countries, and eleven were randomized controlled trials. Sample size ranged from 31 to 2503 participants, and 13 studies included 60% or more women. Interventions were most frequently delivered via print materials and phone (n = 11), compared to internet (n = 3) and other media (n = 2). Every intervention was theoretically framed with the Social Cognitive Theory (n = 10) and the Transtheoretical Model of Behavior Change (n = 6) applied mostly. Individual tailoring was reported in 15 studies. Physical activity levels were self-assessed in all studies. Fourteen studies reported significant increase in physical activity. Eight out of nine studies conducted post-intervention follow-up analysis found that physical activity was maintained over a longer time. In the six studies where intervention dose was assessed the results varied considerably. One study reported that 98% of the sample read the respective intervention newsletters, whereas another study found that only 4% of its participants visited the intervention website more than once. From this review, non-face-to-face physical activity interventions effectively promote physical activity in older adults. Future research should target diverse older adult populations in multiple regions while also exploring the potential of emerging technologies.

## Introduction

The United Nations (UN) reported that in 2012 about 810 million people were 60 years or older. It was further projected that by 2050 the global community will consist of more than two billion older adults [1]. Thus, the World Health Organization (WHO) described the phenomenon of population aging as one of three major factors influencing global health [2], because older age is closely associated with chronic multi-morbidity [3] and high health care costs [3,4].

An effective non-pharmaceutical way to prevent chronic diseases, increase quality of life and promote general health in older adults is through physical activity (PA) [5-7]. Recent evidence suggests that PA is the strongest predictor of healthy aging and lower probability of disability in older men [8]. Further, PA in older age was reported to positively affect leukocyte telomere length [9], the most important marker of biological aging [10]. Active older adults were also found to enjoy up to 3.2 years longer life without cardiovascular disease [11]. Moreover, researchers observed that PA in older age is associated with enhanced brain plasticity [12,13] especially in the hippocampus [14]. The increased hippocampal volume was reported to significantly influence memory functions with improved cognitive performance in older adults [14]. Finally, PA was found to have a direct impact on mental health in older adults because being physically active is associated with reduced risk of dementia, depression [15] and anxiety [16].

Based on this evidence the WHO recommends older adults to be moderately physically active for a minimum duration of 150 minutes throughout the week, or to at least increase PA levels according to individual abilities in order to enjoy general health benefits [2].

Despite the global release of PA recommendations, studies on older adults indicate low participation and a trend of decreasing PA with increasing age [17]. To address the lack of PA, various interventions aimed to increase PA levels in the older adult population have been developed and evaluated. According to the findings of eight reviews, several intervention components seem to be essential for successfully increasing PA participation in older adults: a) home-based or informal interventions, b) individual tailoring and self-monitoring, c) application of theoretical frameworks, d) not in combination with other interventions, e) moderate intensity and low weekly PA frequency, f) simple and convenient lifetime activities, as well as g) low cost [18-25].

Non-face-to-face interventions are conducted with reduced or no in-person interaction between the intervention provider and participants. They present a novel way to promote PA in older adults. These interventions come with considerable administrative as well as logistic benefits in terms of cost-effectiveness, ease of dissemination and outreach. They can also incorporate the components of successful PA interventions listed above [10,26-28]. The increasing use and social integration of modern media among older adults underpins the PA promotion opportunities of non-face-to-face interventions [29]. Older adults are the fastest growing segment of internet users with 53% of older Americans (≥65 years) using the World-Wide-Web or email [30]. Irvine et al. [28] found that 70% of their older adult sample was online on a daily basis. Hence, this age group is increasingly keen to use modern computer technology [31] irrespective of gender [32]. Consequently, these advanced technologies have potential for new PA promotion strategies in addition to traditional and commonly applied telephone and print based programs [28]. However, no systematic review has evaluated PA interventions targeting older adults that were delivered almost entirely without direct face-to-face contact between study participants and the researcher.

Therefore, the aim of this review was to examine the published literature on non-face-to-face interventions designed to promote PA in community dwelling older adults.

## Methods

### Literature search strategy

A systematic search for non-face-to-face PA intervention studies targeting older adults published between 1^st^ January 2000 and 31^st^ May 2013 in the English language was performed using the literature databases Pubmed, Embase, CINAHL, SportDiscus, ScienceDirect and the Cochrane Library. For each database, the search was constructed using relevant medical subject headings (MeSH). The search terms ‘physical activity’, ‘exercise’ and ‘walking’ were combined with the terms for the intervention delivery mode: ‘media’, ‘newspaper’, ‘TV’, ‘television’, ‘radio’, ‘internet’, ‘web’, ‘world-wide-web’, ‘e-mail’, ‘online’, ‘web-based’, ‘website’, ‘PC’, ‘mail’, ‘print’, ‘broadcast’, ‘video’, ‘phone’ ‘telephone’ and with the terms for the target population; ‘older adults’, ‘seniors’, ‘elderly’, ‘retiree’.

The search term “walking” was specifically included because it is a popular form of PA with a high public health impact especially among older adults [33,34].

Reference lists of relevant publications were scanned for eligible articles. Experts in the field of PA research were contacted to obtain additional papers.

### Study selection

**Figure 1:**
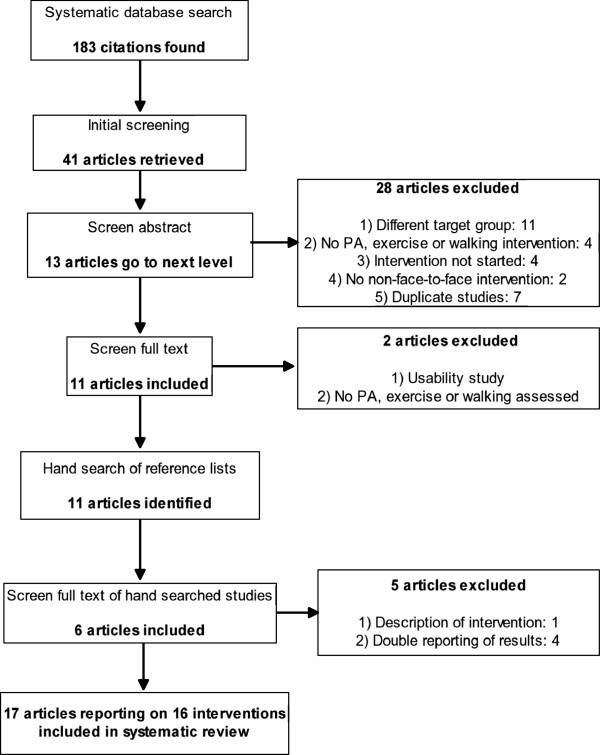
Flow diagram of the search for relevant articles.

The following inclusion criteria were defined before the systematic literature search was performed: a) Study sample consisted of healthy, community dwelling adults aged 50 years or older (because there is no globally accepted definition of older adulthood 50 years was chosen to include relevant studies that have lower cut-offs in terms of age), b) Study implemented a non-face to-face intervention to initiate, increase and/or maintain PA, exercise and/or walking, c) Quantitative data was used to report the effectiveness of interventions. The authors opted against including only randomized controlled trials (RCTs) in order to provide a more comprehensive picture. Unpublished work, review papers, meta-analysis studies and articles that focused on either patients or senior athletes were excluded. When uncertainty about article inclusion occurred the authors came to agreement through discussion. Figure 1 displays the flow diagram of the search for relevant articles.

### Risk of bias assessment

The authors evaluated the risk of bias of included papers independently using the Cochrane risk of bias assessment tool [35]. This instrument assessed the following features: sequence generation, allocation sequence concealment, blinding, incomplete outcome data, selective outcome reporting and other potential sources of bias. Low risk of bias is indicated by answering ‘Yes’ to the respective questions, whereas ‘No’ signifies a high risk of bias. The option ‘Unclear’ indicates a lack of sufficient information for risk assessment or uncertainty about the risk of bias. Although the instrument was specifically developed for RCTs, the Cochrane collaboration suggested that it can also be applied for other research designs [35]. Any discrepancies between the authors regarding the risk of bias were discussed and consensus was reached.

### Data extraction

The following data were extracted from each article for coding using a standardized form: author and country, study focus, participants, study design, theoretical background of the intervention and intervention components, intervention description, outcome measures and outcomes. Data extraction was performed by the corresponding author and checked for accuracy by the co-author.

## Results

The authors identified 183 unique citations through data-base search. After an initial screening 142 records were rejected. Of the 41 remaining articles, 28 were excluded because the described studies did not meet the inclusion criteria. The full text of 2 of the 13 remaining articles was read because the abstract did not provide the necessary inclusion information. These 2 articles were excluded because 1 assessed the usability of a PA intervention and the other did not report data on PA, exercise or walking. After hand searching and forward citation tracking the remaining articles, another 11 papers were added. Of these, 5 were excluded because 1 only described an intervention and 4 presented results that were already reported in the included articles. Results of the study by van Stralen [36,37] were reported in two separate papers, and therefore both papers were included in the analysis. Finally, 17 articles describing 16 intervention-studies were included in the review. The majority of studies were RCTs (n = 11), whereas the remaining studies employed different designs (n = 5) (see Additional file 1 for the risk of bias assessment).

### Study characteristics

All included studies were conducted in developed countries (USA: n = 11, Australia: n = 3, New Zealand: n = 1, Holland: n = 1). The sample size of the studies varied from 31 to 2503, with 13 studies reporting more than 60% female participants. Information on education of the intervention participants was provided in 15 studies. The percentage of participants with at least a college degree (higher education) ranged from 27% to 72.3%. Nine studies employed only one non-face-to-face intervention delivery strategy to promote PA with most studies using print or telephone-based approaches (n = 11). However, internet interventions are emerging (n = 3). Every intervention was theory-guided, with the Social Cognitive Theory (SCT, n = 10) and the Transtheoretical Model of Behavioral Change (TTM, n = 6) the most applied. Other frequently reported intervention components were tailoring (n = 15), goal setting (n = 8), self-monitoring (n = 6) and motivation (n = 6). Self-report instruments to measure PA were used in all 16 studies with the Community Healthy Activities Model Program for Seniors (CHAMPS) being the most frequently applied (n = 5). Additionally, one study used behavioral observations at popular walking sites in order to assess changes in walking frequency in the respective intervention communities [38]. Accelerometer counts complemented self-report data in one study [26]. Thirteen studies reported main PA outcomes in weekly time spent in PA or walking and/or weekly energy expenditure. Eight interventions were short-term and lasted between one week and three months. Long-term interventions (n = 8) lasted up to 24 months. Nine studies conducted post-intervention follow-up analysis, which ranged between 1 and 18 months. Table 1 summarizes important information from each of the interventions.

**Table 1:** Summary of non-face-to-face PA intervention studies targeting older adults

**Print and phone interventions**					
**Reference**	**Study design**	**Demographics**	**Intervention components**	**PA measures**	**Results**
Ball et al. [39]	RCT	66 physically underactive adults (45–78 years, 73% female), Australia	12 weeks print, and print plus phone individual PA counselling; SCT, TTM; self-monitoring, goal setting, incentives, tailored feedback for print plus phone group	CHAMPS: global PA scores, MET-min/wk for PA and walking measured at baseline, 12 and 16 weeks	Significant increase of global PA scores from baseline to 12 weeks maintained after 16 weeks, • significant increase in MET-min/wk maintained after 16 weeks, • significant increase in MET-min/wk for walking from baseline to 12 weeks and from 12 weeks to 16 weeks in print plus phone group
Castro et al. [40]	RCT	140 sedentary adults (50–65 years, 43% female, 15.6 years ±2.7 years education), USA	12 months mail only, and mail plus phone PA maintenance intervention after 12 months PA adoption intervention; SCT; motivation, self-monitoring, relapse prevention and overcoming barriers, tailored feedback	Self-report exercise adherence and activity log: monthly exercise adherence based on prescribed exercise sessions recorded for 12 months	During maintenance period PA levels remained over baseline, • mail only intervention with significantly higher PA maintenance
Greaney et al. [41]	RCT	966 adults (≥60 years, 71.4% female, 12.9 ± 2.9 years education), USA	Written material, newsletters, Expert System Assessment and coaching calls for 12 months; TTM; stage specific tailored feedback, encouragement	YPAS: exercise, household and recreational PA during typical week in previous month, YPAS score (higher score: more active), measured at baseline, 12 and 24 months	No significant change in YPAS scores (baseline: 46; 12 months: 46; 24 months: 47);
Hooker et al. [42]	Community intervention at 13 sites (not randomized)	447 sedentary or irregular active adults (≥50 years, 78.3% female, 27.2% higher education), USA	18 phone calls in 12 months after initial face-to-face meeting where individual PA plan was developed; SCT; tailored support and feedback	CHAMPS: total PA energy expenditure, total hours PA and PA frequency per week measured at baseline, 6 and 12 months	Significant median increase in PA energy expenditure (baseline-6 months: 644 kcal/wk; baseline-12 months: 707 kcal/wk), • significant median increase in total PA hours (baseline-6 months: 2.75 h/wk; baseline-12 months: 3 h/wk), • significant increase in PA frequency (baseline-6 months: 3 times/wk; baseline-12 months: 4 times/wk), • no changes between 6 and 12 months
King et al. [26]	RCT	189 underactive adults (≥55 years, 69.3% female, 16.2 ± 1.9 years education), USA	12 months PA phone counselling by human counsellor, or by computer controlled interactive system supplemented by info mailings and pedometer; SCT, TTM; tailoring, self-monitoring	Stanford 7-Days Physical Activity Recall: energy expenditure and weekly minutes in moderate-vigorous PA; CHAMPS measured at baseline, 6 and 12 months; Accelerometer for 7 d (26% of sample) recording moderate PA	Significantly greater mean energy expenditure and mean PA minutes per week in intervention groups compared to controls (6/12 months), • significantly more days of 30 minutes moderate-vigorous PA per week in intervention groups (6/12 months), • significantly more participants met WHO PA recommendation in intervention groups (6/12 months), • significantly more PA based on accelerometer counts in intervention groups
Kolt et al. [43]	RCT	186 low active adults (≥65 years, 66.2% female, 44.1% higher education), New Zealand	Eight PA counselling calls in 3 months; TTM; individual goal setting, providing knowledge and motivation, problem solving and relapse prevention, tailoring	Auckland Heart Study Physical Activity Questionnaire: PA frequency per fortnight (leisure, walking, occupational, domestic) and number of minutes per time measured at baseline, 3, 6 and 12 months	Significantly more total leisure time PA in intervention group compared to controls from baseline to 3 months (48.9 min/wk, SE 21.6 min/wk), • significantly more total leisure time in intervention group from baseline to 12 months (86.6 min/wk), • significantly more participants in intervention group met PA recommendations after 12 months compared to control group
Lee et al. [44]	RCT	270 inactive adults (65–74 years, 65.3% female, 32.3% higher education), Australia	Interactive booklet and individual PA counselling calls for 12 weeks (five phone calls); participatory action research; tailoring, goal setting, self-monitoring, motivation	IPAQ short form: frequency (days, times) and duration (minutes) of walking and PA per week measured at baseline and 12 weeks	Significant differences between intervention and control group in recreational walking and PA at post intervention, • significant gain in recreational walking and PA (27 minutes/wk) in intervention group
Martinson et al. [27]	RCT	1049 moderately active adults (50–70 years, 72.4% female, 66.7% higher education), USA	7 phone sessions in 6 months, followed by monthly and bimonthly calls in year one and two; control group with information material and 4 newsletters; SCT; relapse prevention, self-management (goal setting, problem solving, identification of barriers, self-monitoring, environmental cues), tailoring	CHAMPS: total kcal/wk, weekly kcal spent in moderate to vigorous PA; meeting PA guidelines measured at baseline, 6, 12 and 24 months	Significantly more participants in intervention group reported more kcal/wk expended at 6 (p < 0.03) and 24 months (p < 0.01) follow-up, • intervention group continued to increase kcal/wk expenditure over 24 months, • intervention group participants reported significantly more kcal/wk expenditure in PA than controls at 6 (p < 0.03), 12 (p < 0.04) and 24 months (p < 0.01), • significantly more intervention group participants maintained PA at 6 (p < 0.001), 12 (p < 0.03), and 24 months (p < 0.001)
van Stralen et al. [36,37]	RCT	1971 adults (≥50 years, 57% female, 52% middle or higher education), Holland	Three computer tailored PA advice letters; or additional environmental focused information on PA opportunities in neighborhood plus access to e-buddy system for 4 months; SCT, I-Change Model, TTM, health action process approach, precaution adoption process model, self-regulation theory, self-determination theory; tailoring	Dutch Short Questionnaire to assess Health enhancing PA: total weekly PA and total weekly PA minutes, compliance to PA guidelines, self-rated PA level measured at baseline, 3, 6 and 12 months follow-up	Significant increase in total PA at 3 months with further increase at 6 months in intervention groups compared to controls, • intervention groups complied with PA guidelines 1.6 times (3 months) and 2.5 times (6 months) more than controls, • insufficiently active
					intervention participants more likely to have initiated
					PA at 3 months than controls with further increase at 6 months, • significant increase in total days/wk of sufficient PA in intervention groups from baseline (4.2 ± 2.2) to 12 months (4.7 ± 2.0) with medium effect sizes, • only intervention with PA opportunities information significantly effective (small effect) in increasing total PA min/wk
Walker et al. [45]	Randomized by site community-based controlled clinical trial	225 rural, irregular active older adults (50–69 years, 100% female, 35% higher education), USA	18 tailored PA newsletters and instructional video versus generic PA newsletters for 12 months; SCT; tailoring, motivation, overcoming barriers, goal setting	Modified 7-Day Activity Recall: daily PA minutes, daily PA kcal expended, weekly time engaged in strength/flexibility training measured at baseline, 6 and 12 months	Intervention and control group significantly increased on all PA measures from baseline to 6 months, • both groups significantly increased weekly stretching and strength exercise from baseline to 12 months, • only tailored group significantly increased daily moderate or higher intensity PA minutes (337.65 ± 675.4 min/wk – 509.88 ± 749.5 min/wk, p < .001)
Wilcox et al. [46]	Community study of previously tested intervention (quasi experiment)	2503 underactive adults (≥50 years, 80% female, 33% higher education) recruited from different sites over four years, USA	Six months phone PA counselling; SCT, tailoring, goal setting, self-monitoring, motivation	CHAMPS: min/wk spent in moderate to vigorous PA, total PA, meeting PA guidelines measured at baseline and 6 months	Significant PA increase and significant increase of participants meeting PA guidelines (p < .001)
**Internet interventions**					
Hageman et al. [10]	RCT	31 inactive adults (50–69 years, 100% female, 51.7% higher education), USA	Three tailored versus non-tailored online newsletters in 3 months; SCT; tailored information based on baseline assessment for one group	Modified 7-day Activity recall: daily energy expenditure, weekly PA minutes measured at baseline and 3 months	Non-significant decrease in energy expenditure (mean decrease of calories expended daily 6.4%) and weekly PA minutes (mean decrease 6.4%) for both groups
Irvine et al. [28]	RCT	405 sedentary adults (≥55 years, 69% female, 82% some college education), USA	12 weeks multiple visit stand-alone internet intervention with text and videos; Theory of Planned Behavior (TPB); goal setting, tailoring	Self-developed tool measuring weekly PA frequency and PA minutes applied at baseline, 12 and 24 weeks	Large PA gains from baseline to 12 weeks (eta square = 0.17), • Medium to large effect sizes for cardiovascular, stretching, strengthening, balance activities and weekly PA minutes from baseline to 12 weeks maintained after 24 weeks
Ammann et al. [47]	Quasi experiment	235 adults (60–89 years, 57% female, 72.3% higher education), Australia	Website for individual PA advice, 1 week online; TPB, TTM; tailored feedback	Active Australia Survey: PA levels (duration, frequency of walking, PA in previous week), total PA minutes, PA sessions measured at baseline, 1 week and 1 month	Significant increase in total weekly PA minutes and PA sessions from baseline to 1 month (327 min ±335 min to 404 min ± 345 min; 8.3 sessions ±7.2 sessions to 10.1 session ±7.6 sessions), • non-significant increase in walking minutes, moderate and vigorous intensity PA
**Other media interventions**					
King et al. [48]	RCT	37 inactive adults (≥55 years, 43.4% female, 16.8 ± 2.2 years education), USA	Educational material, and daily PA feedback delivered via PDA for 8 weeks; or written material only; SCT; tailored PA goal setting and feedback	CHAMPS: minutes/wk spent in moderate to vigorous PA, total PA, meeting PA guidelines measured at baseline and 8 weeks	Significantly higher 8-week moderate PA minutes in PDA group compared to controls (PDA mean = 310.6, SD 267.4 minutes; control mean = 125.5, SD 267.8 minutes; p = 0.048) and caloric expenditure in kcal/kg/wk in moderate PA (PDA mean = 19.1, SD 16.8 kcal/kg/wk; control mean = 7.8, SD 16.8 kcal/kg/wk; p = 0.05)
Reger et al. [38]	2 community longitudinal study (quasi experiment)	463 adults (50–65 years, 68% female, 24.7% higher education), USA	Promoting walking in Wheeling, USA using paid media (newspaper, TV, radio) and public relations events for 8 weeks; TPB, elaboration likelihood model for advertisements	Behavioral observation of walking: counting walkers at popular walking sites; self-developed PA questionnaire: weekly days of brisk walking and moderate to vigorous PA, hours and minutes devoted to activity per day measured at baseline and 8 weeks	Significant (23%) increase in walking in intervention community compared to 6% decrease in comparison community (p < 0.0001, OR = 1.31, 95% CI = 1.14-1.50), • 32.2% met walking guidelines (150 minutes/wk) in the intervention community compared to 18% in comparison community (p < 0.05, OR = 2.12, 95% CI = 1.41–2.24), • no significant effects in other PA

### Effectiveness of interventions

Of the 16 studies, 14 reported significant improvements in PA over the respective study periods (1 week to 24 months). No changes in PA measured with the Yale Physical Activity Survey (YPAS) was observed by Greaney et al. [41] where PA scores between baseline, post-intervention and 12 months follow-up did not vary. Only the internet-based study by Hageman et al. [10] reported a non-significant decrease of PA in terms of daily calorie expenditure and time spent in moderate or greater PA over the previous week.

PA levels were maintained after the intervention stimulus was removed in all but one study [41] that collected follow-up data. Ammann and colleagues [47] conducted a short, one week internet intervention (website), and reported that the positive effects sustained after one month follow-up. Strong PA maintenance was also reported by Castro et al. [40]. The researchers found that 73% of a mail and 57% of a mail plus phone intervention maintained PA levels over the 12 months post-intervention follow-up period. Martinson et al. [27] conducted follow-up analysis after a 6-month telephone intervention and found that PA maintenance of the intervention group increased from 43.4% (6 months follow-up) to 50.1% after 18 months without the intervention stimulus.

### Intervention dose

Intervention dose (completed phone calls, website visits, use of written material) was assessed in six studies. The study by van Stralen et al. reported that 98% of the print intervention participants read all three letters that were sent during the 4-month intervention period [37]. King et al. [26] conducted a 12-month phone intervention and recorded a mean of 12.5 completed calls per participant. Web-based studies (n = 3) showed differing doses. Hageman et al. [10] found that 83% of the study participants read all three online newsletters, whereas Ammann and colleagues [47] stated that only 4% of their sample visited the intervention website at least twice within one week. The study by Irvine et al. [28] reported a mean of 15.2 PA website visits (±9.2 visits) during the 12-week intervention. As a result of an extensive 8 weeks paid media PA intervention (TV, radio, newspaper, website, events) 90% of the study community was aware of the campaign [38].

## Discussion

The aim of this systematic review was to determine how far non-face-to-face PA interventions in older adults can lead to PA adoption, increase and/or maintenance. The results suggest that the respective interventions are effective in enhancing PA participation in healthy, community dwelling older adults.

Only two studies presented contradictory results. Greaney et al. [41] reported no change in PA levels after a 12-month print and phone intervention. However, the study sample was already reasonably active and further increase in PA was less likely [49]. Further, no measure of the intervention dose, literacy or cognitive status of the participants was undertaken. Hence, it is unclear whether the study participants read and understood the printed material [13]. The study by Hageman et al. [10] reported non-significant decreases in PA time and energy expenditure after a web-based newsletter intervention. This seems surprising because the perception of barriers decreased and PA self-efficacy increased. Though, a considerable number of research studies have shown that changes in motivational constructs do not necessarily lead to actual behavior [50,51]. A study by Sheeran [52] found inclined abstainers (non-actors with behavioral intentions) to be most responsible for not showing the respective behavior. Finally, this was the first internet-based PA intervention targeting older adults. Hence, it can be inferred that older adults were not as technologically literate compared to today [29] and therefore did not benefit as much as participants recruited for more recent interventions [28,47].

### Intervention components

Although it is difficult to compare heterogeneous PA interventions that applied disparate methodologies it is possible to identify some interventional features that may influence PA participation in older adults. As stated in previous reviews [22,23] and mirrored in this review a theoretical framework seems to be an essential interventional element for PA interventions in older adults. Yet, although all the interventions applied at least one theory, the effects on PA outcomes cannot be inferred. Moreover, Prestwich et al. [53] conducted a meta-analysis and reported that theory application in health behavior interventions, including PA, has only minimal effects on the respective behavioral outcomes. Small effect sizes were also found for theoretically framed internet interventions targeting PA [54]. These results were largely drawn on the basis of SCT and TTM, the same theories that were frequently applied in the studies included in the present review. Therefore, future intervention studies might benefit from applying theories that go beyond cognitive, social and emotional constructs (like in SCT and TTM).

All studies except one [38] applied individual tailoring strategies. Tailoring can be accomplished in various ways and was found to increase the likelihood of successful health interventions because it increases the perception of personal relevance for intervention participants [55]. According to a review by Lustria et al. [55] there are three tailoring strategies that can be applied in intervention studies: personalization, feedback and adaptation. The first two are common, but the last strategy requires more sophisticated procedures because tailoring content needs to be adapted to changing cognitions and behaviors during the intervention. The effects of tailoring versus non-tailoring in terms of PA participation was assessed in three of the reviewed studies. Walker et al. [45] and King et al. [48] incorporated all three tailoring strategies and reported that tailored interventions were more effective in increasing PA compared to non-tailored interventions. Hageman and colleagues [10] used only baseline information for tailoring and did not adapt the content during the 3-month online newsletter intervention. The decrease in PA after the intervention in both study groups (tailored and non-tailored) suggests that extensive tailoring leads to increased intervention effects. Though, more research is needed to gain further insights.

Intervention delivery was mainly by print and phone but internet-based interventions have recently received more research attention and were found to be an effective and economically viable way to influence PA behavior in older adults [28,47]. However, web-based interventions might systematically exclude populations with lower education and less income who have limited computer and internet access. This is critical because these populations are usually also less active compared to people in higher socio economic strata [56]. Ammann et al. [47] reported that 73% of the intervention participants in their study had higher education levels. Similarly, Irvine et al. [28] recorded that 82% of their sample had at least some college education. Researchers of both studies acknowledged that their samples were unlikely to be representative of the general older adult population and that the positive study results must be viewed with caution. In comparison, Hageman et al. [10] recruited a more balanced sample in terms of education (51.7% with higher education) and reported pessimistic intervention results. Overall, these studies suggest that print and phone interventions might remain a valid intervention strategy to increase PA in older adults with low social economic status.

### Outcome measures

A diverse range of self-report PA measures was applied across the studies of the current review. Self-report instruments come with general limitations in terms of misreporting and lack of sensitivity in detecting certain PA changes. According to a recent review, results from PA questionnaires for older adults showed only low to medium correlations with accelerometer counts (median: Spearman r = 0.4, Pearson r = 0.345) [57]. Despite these limitations, the use of self-report measures is widely accepted because of cost and logistic benefits compared to objective instruments [19]. Though, the use of objective measures like accelerometers is desirable in order to provide a more precise picture of the effects of non-face-to-face PA interventions in older adults.

Only six studies reported on the dose of the intervention stimulus. According to Ammann et al. [47] a mere 4% of the study sample visited the study website at least twice during the one week intervention period and participants spent just 16.3 minutes reviewing the content. However, PA increased significantly. In contrast, 83% of the participants in the study by Hageman et al. [10] read all the newsletters sent, but PA scores decreased insignificantly. Even though other studies indicated that higher stimulus dose leads to enhanced intervention effects [28,58] more research is needed to reach clear understanding specific to non-face-to-face interventions.

### Limitations

This review was limited to English language articles. Therefore, evidence from studies published in other languages might have been missed. Further, all included studies were conducted in developed countries (Australia, Holland, New Zealand and United States). Hence, the results of this review are not globally generalizable. Nonetheless, they can serve as a guide for region-specific studies that aim to explore the effectiveness of non-face-to-face PA interventions targeting older adults in various contexts and conditions.

Finally, it is worth noting that 14 of the 16 non-face-to-face PA studies reported positive intervention effects. It is likely that studies that had negative or null findings were either not published or were published as grey literature. This publication bias (publication associated with trial findings) is common in health research [59]. A systematic Cochrane review underpinned this notion and suggested that positive findings were more likely to be published than trials with negative or null findings (OR 3.90, 95% CI 2.68 – 5.68) [59]. This systematic bias threatens the validity of reviews in general. Therefore, future review authors might consider looking for unpublished literature. Although these sources might lack scientific rigor, they can be useful in drawing a more realistic conclusion about intervention effects. Registering trials before the final results are known might eradicate this bias entirely.

Despite these limitations this review has the advantage of using a systematic rather than a narrative approach and constructed eligibility criteria that incorporated studies with various research designs and objective interventional outcomes. It is the first review to report on PA interventions targeting older adults that were conducted with reduced or no in-person interaction between intervention provider and study participants. Thus, the findings can serve as a baseline for future research.

## Conclusions

It is widely accepted that PA is a viable method to enhance overall health and well-being in older adults and therefore reduces the economic burden caused by aging-specific health conditions. The implementation of non-face-to-face PA interventions that come with significant administrative and economic advantages appears to be an effective approach towards adoption, increase and maintenance of PA in older adults. This review reports positive short- and long-term outcomes of non-face-to-face PA interventions and therefore underpins its relevance for community dwelling older adults. The authors recommend further research on older populations who are living in residential aged care facilities or other forms of supported accommodation. Here non-face-to face PA interventions might need to be complemented with in-person contact sessions to account for elevated individual care needs. Furthermore, researchers are encouraged to examine the effects of non-face-to-face PA interventions in multiple regions to enhance the generalizability of the study results. Finally, with the increasing competence of older adults in using new technologies, the use of smartphones for PA interventions should be explored in future research.

## Abbreviations

UN: United nations; WHO: World health organization; PA: Physical activity; MeSH: Medical subject headings; RCT: Randomized controlled trial; SCT: Social cognitive theory; TTM: Transtheoretical model of behavioral change; CHAMPS: Community healthy activities model program for seniors; MET: Metabolic equivalent; YPAS: Yale physical activity survey; SE: Standard error; IPAQ: International physical activity questionnaire; TPB: Theory of planned behavior; PDA: Personal digital assistant; OR: Odds ratio; CI: Confidence interval.

## Competing interests

The authors declare that they have no competing interests.

## Authors’ contributions

AMM developed the concept and design of the study, provided the systematic literature research, extracted the data and drafted the original manuscript. AMM & SK contributed to the interpretation of the data and provided critical revisions to the manuscript. Both authors have read and approved the final manuscript.

## Supplementary Material

Additional file 1 Risk of bias assessment (+: low risk of bias; -: high risk of bias; ?: unclear risk of bias; *: no RCT). Click here for file
