# Supplementary material for: Non-face-to-face physical activity interventions in older adults: a systematic review
Source: Int J Behav Nutr Phys Act. 2014 Mar 10;11:35. doi: 10.1186/1479-5868-11-35 (PMC4008359; doi:10.1186/1479-5868-11-35)
Supplement: Additional file 1 — Risk of bias assessment (+: low risk of bias; -: high risk of bias; ?: unclear risk of bias; *: no RCT). [file 1479-5868-11-35-S1.docx]

**Risk of bias assessment**

|  | Was the allocation sequence adequately generated? | Was allocation adequately concealed? | Was knowledge of the allocated intervention adequately prevented during the study? | Were incomplete outcome data adequately addressed? | Are reports of the study free of suggestion of selective outcome reporting? | Was the study apparently free of other problems that could put it at a high risk of bias? |
| --- | --- | --- | --- | --- | --- | --- |
| Ammann et al. [47]* | - | - | - | + | + | - |
| Ball et al. [39] | + | ? | ? | + | + | + |
| Castro et al. [40] | + | ? | + | + | + | + |
| Greaney et al. [41] | ? | ? | + | + | + | - |
| Hagemann et al. [10] | ? | ? | ? | + | ? | + |
| Hooker et al. [42]* | - | - | - | + | ? | - |
| Irvine et al. [28] | + | ? | - | + | + | - |
| King et al. [48] | ? | ? | ? | + | ? | - |
| King et al. [26] | + | + | + | + | + | ? |
| Kolt et al. [43] | + | + | + | ? | + | ? |
| Lee et al. [44] | ? | ? | - | ? | ? | - |
| Martinson et al. [27] | + | + | + | + | ? | ? |
| Reger et al. [38]* | - | - | - | ? | ? | - |
| Van Stralen et al. [36,37] | - | - | ? | + | + | ? |
| Walker et al. (2009) [45]* | - | - | ? | + | ? | ? |
| Wilcox et al. (2008) [46]* | - | - | - | + | ? | ? |

**Additional file 1.** Risk of bias assessment (+: low risk of bias; -: high risk of bias; ?: unclear risk of bias; *: no RCT)
